# A review: the effect of the microporous support during interfacial polymerization on the morphology and performances of a thin film composite membrane for liquid purification

**DOI:** 10.1039/c9ra07114h

**Published:** 2019-11-01

**Authors:** Feng Liu, LanLan Wang, Dawei Li, Qingsheng Liu, Bingyao Deng

**Affiliations:** Laboratory for Advanced Nonwoven Technology, Key Laboratory of Eco-Textiles, Jiangnan University, Ministry of Education Wuxi 214122 People's Republic of China bydeng@jiangnan.edu.cn

## Abstract

The thin film composite (TFC) membrane prepared by interfacial polymerization (IP) on porous supports is currently one of the most efficient technologies for brackish water purification and seawater desalination, including reverse osmosis (RO), forward osmosis (FO), and nanofiltration (NF). Over the past decades, there have been intensive and continuous efforts in research of polyamide layers, while there is little information in the literature about the impact that physical–chemical properties and structure of support membranes have on the formation of composite membranes. This paper reviews the recent research progress of the supporting membrane, comprehensively summarizes the support role in polyamide formation, and provides good insight into TFC membrane research and development. In addition, we discuss several types of polymer supporting membranes and related modification methods to explore the appropriate supporting membrane for enhancing TFC membrane performance and extending the applications in the future.

## Abstract 2

The thin film composite membrane prepared by interfacial polymerization on porous supports is currently one of the most efficient technologies for brackish water purification and seawater desalination, including reverse osmosis, forward osmosis and nanofiltration.

## Graphical abstract

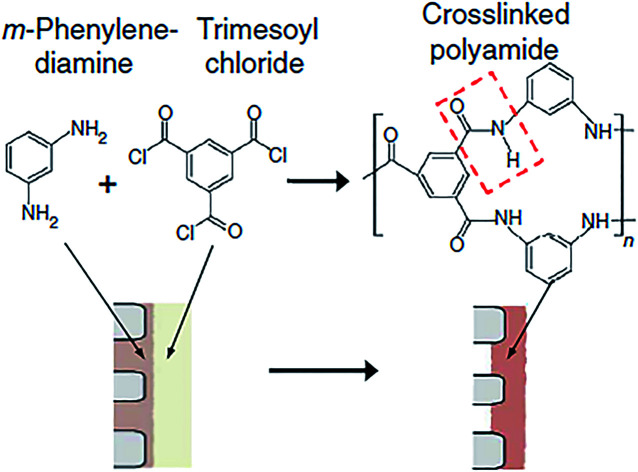

## 1. Introduction

Water scarcity and water pollution have become increasingly important problems as society develops and population booms. The drinking water and agricultural irrigation demand have been constantly growing. Seawater desalination and sewage purification have drawn great attention as a new water resource.^1–3^ In the past few decades, membrane separation processes have developed rapidly, and became a dominant technology.^4^ The membrane separation process is one of the most effective, convenient and promising methods used in water treatment systems. At present, existing various desalination technologies like forward osmosis (FO), nanofiltration (NF) and reverse osmosis (RO) membranes, are used for purification and filtering of sewage or brackish water to obtain clean water for production and daily life. RO is the most energy-efficient technology for seawater desalination also is the benchmark for evaluation of a new desalination technology. The water is treated by RO membrane which can almost remove more than 95% of soluble particles in the water, and the purified water can reach the standard of direct drinking. FO process using membrane based technology has attracted considerable attention among membrane scientists as a potential desalination process. Because of the energy consumption in FO is considerably lower and applied low operating pressure is reduced fouling tendency.^5–11^

TFC membrane was developed for tens of years, at present, there are many ways for the preparation of TFC membrane, include interfacial polymerization (IP), coating technology and post-modification treatment. Sun *et al.* prepared a kind of composite membrane with a mussel-inspired polydopamine coating layer based on polyimide support. Cross-linked hyperbranched polymer networks have been developed as the active layer for TFC membrane. The PA TFC membrane fabricated *via* IP was used widely in sewage water purification, as well as seawater and brackish water desalination. RO membrane technology was developed in middle of 20^th^ century. After half a century, there are two types of major RO membrane, cellulose acetate (CA) membrane and aromatic polyamide (PA) membrane. Relatively speaking, PA TFC membranes have many outstanding advantages, for example, wide range of applications, low environmental requirement, stable structure, pressure resistance and less biological pollution. In addition, conventional TFC membranes are composed of a thin active layer on top of a thick support layer, because of TFC membrane layer structure, ultrathin and high crosslink PA layer formed on porous substrate, good water osmosis flux and high salt retention of PA TFC membrane was fabricated.^12^ For the past few years, interfacial polymerization, a predominant method for the fabrication of TFC RO membrane, is now being explored as FO membrane and NF membrane. Generally speaking, the PA active layer of TFC membrane is prepared on the top side of porous substrates *via* interfacial polymerization (IP) of *m*-phenylene diamine (MPD) in aqueous solution and trimesoyl chloride (TMC) in organic solution. TFC membranes are flexible, and the chemistry and performance of both top-layer and support can be independently manipulated to affect the overall membrane performance to achieve desired selectivity and permeability while offering excellent mechanical strength and compression resistance.^13^ However, most of researchers think that PA active layer is the deciding factor for the separation and water osmosis property of TFC membrane, and there are most information in literature about the impact that physical–chemical properties of active layer have on the formation of composite membranes. There are few reports on the impact that physical–chemical properties of support layer on the formation of composite membranes.^14–18^

It is well acknowledged that by employing IP method, the properties of support layer and active layer can be individually tailored and optimized to achieve desired water permeation and solute separation rate. The porous support membrane not only provides a mechanical layer on which to build the composite structure, but the morphology and chemistry of support may also influence the formation of the ultrathin polyamide layer.^19^ As is known to all, the physicochemical properties of the porous support such as hydrophobicity, porosity, pore size and roughness are important factors shaping the morphology of PA TFC membrane and showed significant influences on formation of selective layers.^5,20–22^ Until now, several research groups have started studying the effect of support performance on PA layer formation to gain a better understanding on the formation mechanisms between the active layer and the substrate. In spite of this, these literatures focused on the effect of single factor or double factors of support on interfacial polymerization, lack of comprehensive and detailed explanations of the various impact factors of support. We reviewed the mechanism of action of porous support layer in interfacial polymerization by concluding the recent research progresses of the TFC membrane in order to provide an insight and comprehensive understanding of the future development, particularly the selection of appropriate supporting materials of TFC membrane with improved performance.

## 2. Mechanism of formation, structure and application of the TFC membrane

**Fig. 1:**
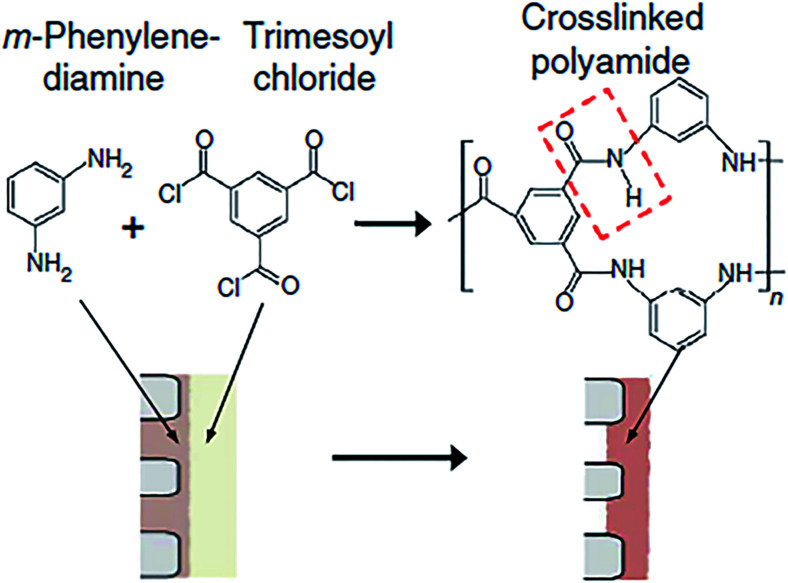
Standard polyamide polymerization derived from MPD and TMC solution on porous support.^2^

The TFC polyamide membrane consists of three layers: a polyester non-woven fabric acting as the structural support (120–150 μm) to offer excellent mechanical strength, a porous interlayer (about 50 μm, with pore size between 20–100 nm) to withstand high pressure compression, and an ultra-thin PA active layer (about 200 nm, with pore size around 0.5 nm) by interfacial polymer on porous support to achieve high salt rejection. The standard interfacial polymerization mechanism is shown in Fig. 1. Each layer of TFC membrane is not only independently controlled and optimized composite membrane performance, but can also synergistic effect between them to charge properties and applied environment. Selective permeability and water flux are the important indicators to evaluate composite membrane properties, it is up to the thickness and the degree of crosslinking of PA film. High crosslinking degree and thinner PA layer lead to a high water flux and salt rejection, whereas low crosslinking degree and thick PA layer result in low water flux and poor salt rejection.

For past few decades, some research groups fixed their eyes on membrane formation mechanism, and selected appropriate polymeric materials to enhance the functionality and durability of membrane. Recently, some researchers started to pay attention to the role of support performance in interfacial polymerization. Their results showed that the structure parameters (pore size, porous, roughness, and surface hydrophilicity or hydrophobicity) affected the morphology and property of PA membrane significantly in the process of condensation. Furthermore, those parameters affected indirectly the water osmosis flux and salt rejection of TFC membrane. Condensation polymerization between MPD with TMC occurred on porous support surface. First, the support was soaked in MPD aqueous solution for a few minutes and the excess aqueous solution was removed with an air knife or filter papers. The support impregnated with the MPD solution contacted with the organic TMC solution for a short time to produce a highly cross-linked, network-structure PA active layer. The nuclear reaction was the residual of MPD solution react with TMC solution in a short period, on the surface and inside the pores of support. Thus, the pore size, porous, roughness, hydrophilic and hydrophobic of support surface play important roles in the IP process. The physical–chemical properties of support surface and pore morphology controlled the aqueous solution residual volume in pore and surface, and further determined the thickness and crosslinking degree of PA layer.^23–25^ The support membrane had an important role as it functioned as a container for one of the precursors, and co-defined the interface where the interfacial polymerization reaction would occur.

## 3. The effect of substrate on TFC membrane

### 3.1 The polymer type and forming method of porous support layer

**Fig. 2:**
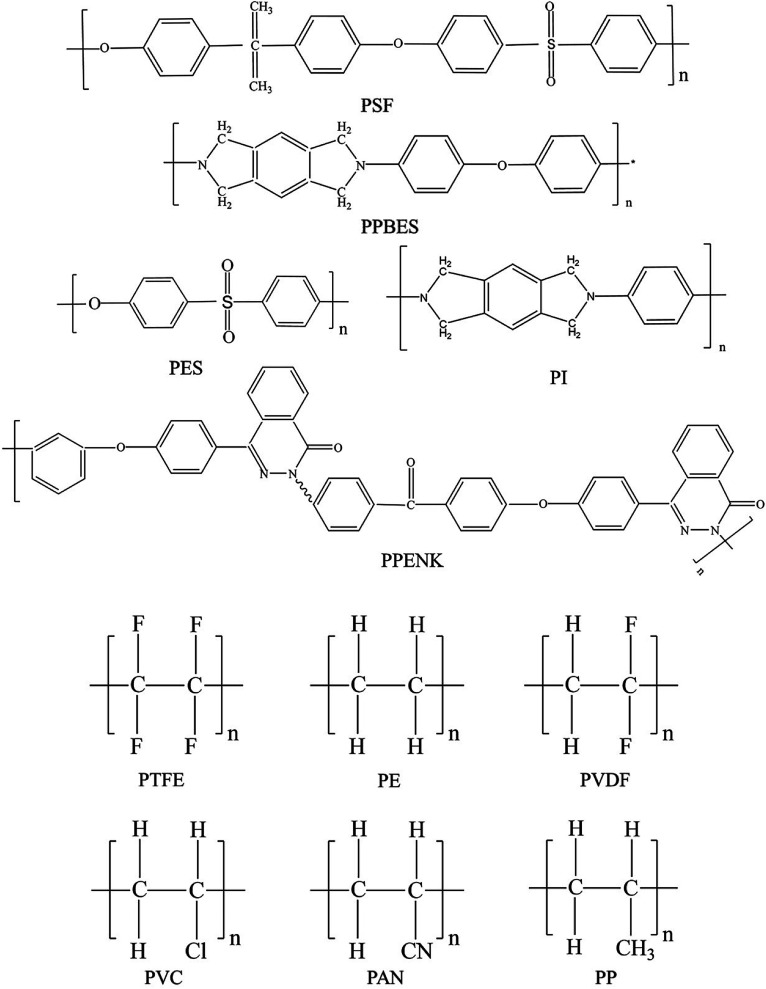
The chemical structure of commonly used support polymers for thin film composite membrane.

The development of multilayer TFC membrane is a major breakthrough in the field of membrane science and technology. The TFC polyamide membrane *via* interfacial polymerization of MPD and TMC on polysulfone (PSU) porous support is commercially the most successful membrane. Polysulfone and polyethersulfone (PES) membranes fabricated by phase inversion method has been widely used as commercial TFC support membrane material due to good thermal resistance, decent chemical stability and easy fabrication. In addition, PSU support is relatively hydrophilic, which is suitable for the interfacial polymerization of PA in aqueous solution.^26–29^ The support substrate layer based on PSU, however, has relatively low mechanical strength and poor resistance to chemicals such as aromatic hydrocarbons, ketones, ethers, and esters. Moreover, PSU raw materials are too flexible to be held in a specific apparatus. Thus, scientists keep exploring new superior support materials with comprehensive performance, and a variety of polymer materials have been applied to fabricate porous support of TFC membrane. Common polymeric support materials include poly(ether sulfone) (PSF), polyacrylonitrile (PAN), polypropylene (PP), polyvinylidene fluoride (PVDF), polytetrafluoroethylene (PTFE), polyimide (PI), and poly(arylene ether nitrile ketone) (PPENK). The chemical structure of a commonly of porous support polymers shown in Fig. 2. These porous membranes were fabricated mainly *via* the phase inversion method, and a few membranes were prepared *via* the electrostatic spinning method. Electrostatic spinning nanofibrous support has several unique features. For example, the ultrahigh surface porosity is close to 80% or higher, the surface pore structures are inter-connected throughout the membrane without dead-pores, and the surface is smooth enough to support the active layer while the nanofiber deposition is uniform.

#### 3.1.1 Porous PAN support

PAN polymer material is commercially widely used as ultrafiltration membranes and other applications because it has good thermal stability, good resistance to solution, and good resistance to multiple chemicals. PAN polymer is relatively hydrophilic compared with other polymeric materials. Therefore, PAN membrane was prepared by conventional casting method or electrostatic spinning method, which is useful in UF, or NF and RO process as well. However, NF and RO membrane are very difficult to be prepared from PAN by the phase inversion method, due to the large shin pore size of PAN membrane. In order to make PAN casting membrane applied in TFC membrane, some investigations modifying PAN membrane by plasma grafting have been reported. Yang *et al.*^30^ reported a new PI TFC membrane for solvent resistant nanofiltration prepared on hydrophilic PAN ultrafiltration membrane *via* the IP process and subsequent imidization process. Klaysom *et al.*^31^ prepared PAN porous support membrane *via* phase inversion method and the PAN membrane hydrolyzed with NaOH before performing the interfacial polymerization. The cyanide group of the PAN support was converted to a carboxylic group to help strengthening the interaction between the amide active layer and the support layer by forming ionic and covalent bonds. Modified TFC-PAN membrane showed significant enhanced water performance and almost maintained a similar salt rejection. Electrospun PAN nanofibrous scaffolds as support layers have been prepared by Yoon *et al.*^32^ as a new thin film nanofibrous composite (TFNC) membrane by IP of polyamides containing different ratios of piperazine and bipiperidine. Compared with the commercial PAN/UF support with the same barrier layer coating, the TFNC membranes exhibited >2.4 times more water osmosis flux than the conventional TFC membranes maintaining the same rejection rate (about 98%). The improved water flux was the result of large open pore structure, the low hydraulic resistance of the nanofibrous support, and the interface region between the nanofiber and IP matrix that enhanced the water transportation capability.

#### 3.1.2 Fluorinated polymers porous support

Fluorinated polymers such as polyvinylidene fluoride (PVDF) and polytetrafluoroethylene (PTFE) are used widely in various fields due to their chemical stability, thermal resistance, and good mechanical strength, as well as toughness and corrosion resistance.^33–38^ PVDF polymer is used as the base polymer for preparation process of NF, MF and UF membranes main *via* thermal induced phase, no-solution induced phase and electrospun spinning methods. PTFE can be well processed to porous fibers or thin membranes *via* solvent-free melt spinning or extrusion and post-stretching method. The formed membrane has good flexibility, uniform pore size distribution, and high porosity. PTFE membrane has found its peculiar importance in water treatment. Based on the advantages of fluorinated polymers, some researchers that search for superior comprehensive performance of support materials have gradually to shift their attention to PVDF and PTFE materials. However, the intrinsic hydrophobicity of PVDF and PTFE poses a great challenge to the application as support layer of TFC membrane, due to non-polar linear molecular configuration of C and F atoms. Surface hydrophilic modification is a promising viable solution. Coating hydrophilic materials on the membrane surface and blending hydrophilic materials in doping solution are two major ways.

#### 3.1.3 Other polymeric material of support

In addition to the porous support polymeric materials mentioned above, some materials of special structures also were gradually brought into focus to obtain high water permeate flux, high salt rejection, and more stable active layer. Conventional TFC RO membrane was designed to polycondensed MDP and TMC on relatively hydrophobic support layer due to RO the water first permeates the active layer by a solution-diffusion mechanism and then percolates through the pores of support layer by hydraulic, therefore, the support layer should be thick enough to withstand high pressure. Pan *et al.* prepared a novel TFC membrane on surface hydrophilic-modified PP membrane. The PP membrane was hydrophiliced *via* UV-induced grafting of acrylic acid (AAc).^39^ Gorgojo *et al.*^40^ fabricated PA-TFC membrane on highly solvent stable cross-linking polyimide support and used an activating solvent as a strategy to increase water flux (from initial 0.2 L m^−2^ h^−1^ bar^−1^ to 1.6 L m^−2^ h^−1^ bar^−1^) and NaCl rejection from initial below 90% to a maximum value of 94%. Ba and Economy^41^ fabricated a thermally stable TFC RO membrane using polyimide support layer. The permeated flux of 2.0 g L^−1^ NaCl solution rose from 0.74 m^3^ m^−2^ day^−1^ to 3.95 m^3^ m^−2^ day^−1^ when the test temperature increased from 25 °C to 95 °C. Yao *et al.*^42^ designed polyvinyl chloride (PVC) support, the TFC membrane formed thereon display a thicker rejection layer with greater crosslinking density. However, in forward osmosis (FO) and pressure retarded osmosis (PRO) process, the support layer should be wetted to ensure adequate adhesion between the polyamide layer and substrate, so support layer have a relatively hydrophilic surface is desirable. Alsvik *et al.*^43^ demonstrated that it was possible to coat a hydrophilic support membrane given enough functional/reactive groups on the surface of the hydrolysis cellulose acetate (CA) support membrane by hydrolysis. The functional/reactive groups first reacted with TMC produced ester bond, then contacted with MPD to react. Finally, soaking in the TMC organic solution to formed PA active layer by IP. In this way, covalent bonds between the support layer active layer were formed and the connection was stable, as well as water fluxes increase from 0.0657 m^3^ m^−2^ day^−1^ to 0.0406 m^3^ m^−2^ day^−1^ at 1.3 Mpa differential pressures. A thermally stable TFC NF membrane was prepared form piperazine (PIP) and TMC on hydrophilic poly (phthalazione ether nitrile ketone) (PPENK) UF membrane by IP method, the flux of membrane was increased about four times when the operation temperature increased from 20 °C to 80 °C. Besides, Hu *et al.*^44^ also investigated the effects of changing monomers concentration, reaction time and organic solvents on the performance of TFC membranes.

### 3.2 The effects of the pore size and porosity of support on PA active layer performance

**Fig. 3:**
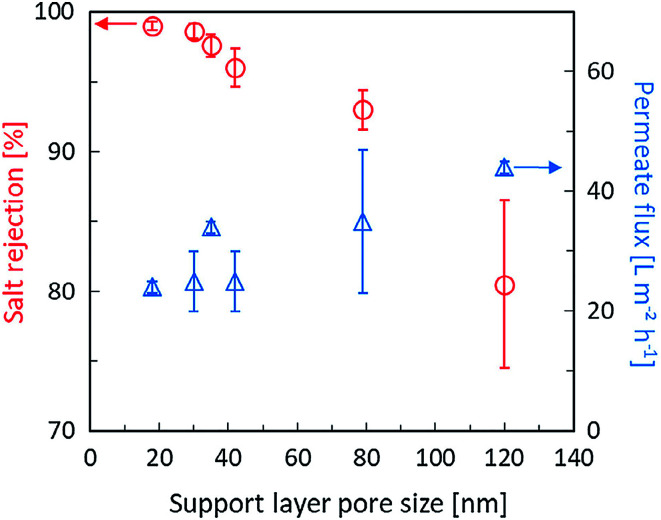
The effect of average pore size of PSF support layers on water flux and rejection of TFC RO membrane at seawater desalination condition.^46^

**Fig. 4:**
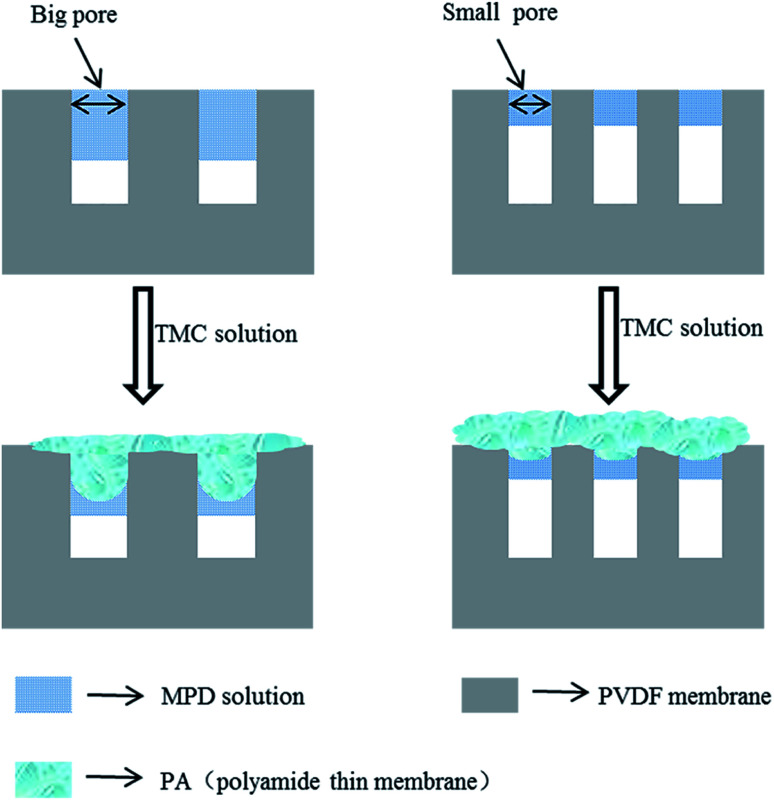
The effect of the pore size of support membrane on PA thin film formation.

By a number of studies and literature reading, we known that the formation mechanism of PA layer during IP process. When the TMC organic solution is poured over the support with an adsorbed aqueous phase containing MPD monomer, MPD molecules migrate to the interface and react with TMC. The reaction mainly takes place in the organic phase and MPD aqueous phase migrate way is convection (diffusion and advection) due to the surface tension gradients between in the two phases.^31,45^ Because of the porous structure of support membranes, most of aqueous solution located in pores. Therefore, the pore size plays a crucial role in governing the migration way of MPD, the support with small pore diameter, where the aqueous phase is thin and diffusion dominates in convection caused “nodular” structure to be formed. The IP reaction produces smooth surface, because diffusion processes have no favoring to form the large PA structure. However, in the case of the support with big pore diameter, there are more and thicker MPD solution adsorbed in pores, and perturbations are promoted by the intense movement of MPD towards the organic phase. During this process, advection plays a dominating role. The reactions are continuously evolving between unreacted TMC and MPD and the early formed domains are pushed and bent to form a rough “ridges and valleys” structure. The pore size not only influences the morphology of PA layer, but also greatly influences the selective properties of PA layer. The PA crosslinking degree of PA is responsible for salt rejection, and higher crosslinking degree corresponds to a higher rejection. The small pore is in favor of forming high crosslinking degree PA, because there are less PA layer inner pores. The structure formation mechanism is believed to cause defects in the inner pore PA layer and results in a decrease in salt rejection. Smaller pores also lead to thicker PA layer on the support surface and the possibility of forming defects lower. Sharabati *et al.*^46^ investigated the impact of support layer pore size on active layer polymerization and seawater desalination performance. Six different TFC membranes with support layers having average pore sizes ranging from 18 nm to 120 nm were fabricated, showing that rejection decrease from 99.0% to 80.5% and the water flux increased. Detailed change was described in Fig. 3. Singh *et al.*^47^ investigated the effect of the pore size of support on the thickness and selectivity of PA layer by coating PA over two PSF membranes that had average pore size distributions of 70 nm and 150 nm. Smaller pore size distributions of substrate was found to have superior salt rejection efficiency compared to bigger pore size distribution, mainly due to significant increase in skin layer thickness following a reduced penetration of PA into pore of substrate, as illustrated in Fig. 4. Although the larger pore size distributions increase PA layer thickness lead to higher salt rejection, weaken the adhesion properties between active layer and support.

The pore size of support layers are relative, and the most optimal pore size of different polymer support layer is discrepant. Choosing an appropriate pore size of the support is crucial to prepare superior performance of TFC membrane. Generally speaking, support layers have average pore sizes ranging from 20 nm to 500 μm. On the support with small pore size of form PA layer have better selectivity. Support layers have average pore sizes ranging from 20 nm to 100 nm, on the support with large pore size form PA layer have better selectivity.

**Fig. 5:**
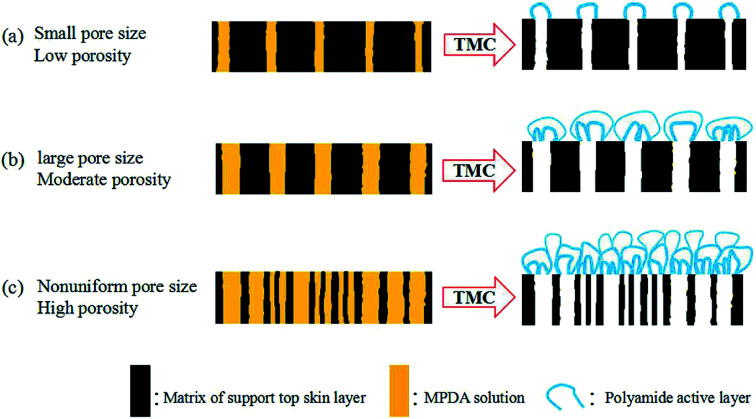
A speculative model for the formation of interfacial polymerized MPDA-TMC films on supports with varied surface pore size and porosity.^48^

According to literatures and studies reported, we learned that not only the pore size of support layer, but also the surface porosity of support affected the growth of active layer, and significantly the performance of TFC membrane. For the same polymeric support layer, on the support with high surface porosity, the produced active layer shown high cross-linking degree, more pronounces surface “ridge-and-valley” structure and higher thickness. On the contrary, on the support with low surface porosity, the produced active layer shown low cross-linking degree, less pronounces surface “ridge-and-valley” structure and lower thickness. Zhang *et al.*^48^ investigated the impact of support surface pore characteristics (pore size and porosity) on the active layer formation process, analyzed the formation mechanism of PA film and proposed a speculated model to outline the interdependence, the speculative model shown in Fig. 5. In IP process, the porosity and pore size of support controlled the amount of residual TMC solution on the support surface. In the case of supports with small pore size and low porosity, a relatively thin PA layer could be obtained consisting of PA bubbles with small size and sparse. In supports with large pore size and moderate porosity, the growth tendency of PA bubble was significantly promoted. In supports with large pore size and high porosity, PA layer constructed by packing PA bubbles closely with complex multilayer structure shown the highest thickness and tightness.^49^

### 3.3 The effect of the hydrophilic and hydrophobic of support on PA active layer performance

**Fig. 6:**
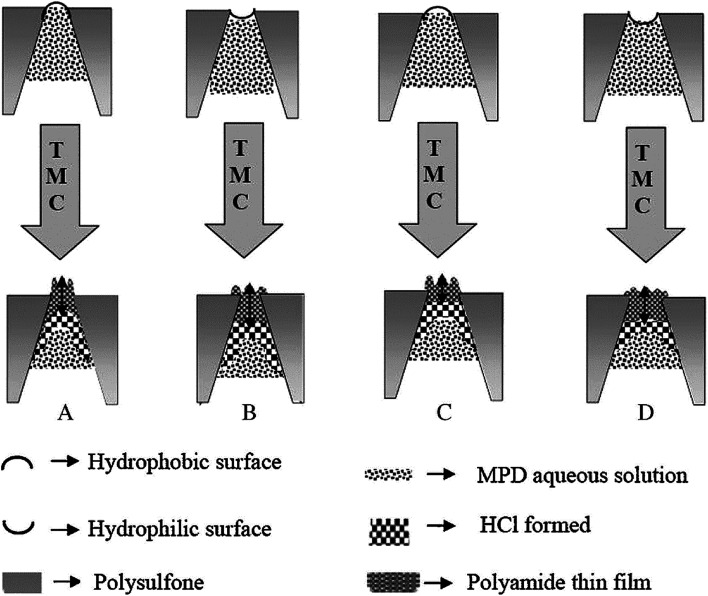
The impacts of PSF support membrane hydrophilic surface and hydrophobic surface on PA TFC membranes formation.^27^

In general, most successful TFC RO membranes have a relatively hydrophobic support to produce high salt rejection PA thin film. However, a more hydrophilic support layer is desirable in PRO process and FO process as such supports will have less internal concentration polarization and better water flux. PVDF, PTFE, PP and others hydrophobic microporous membrane were prepared as supports need a hydrophilic modification to provide good compatibility between support and active layer in IP, due to their inherently hydrophobicity.^50,51^ In supports with hydrophilic surface, more MPD aqueous solution permeated into pores. Furthermore, after the support containing MPD aqueous solution was dried, the aqueous MPD solution meniscus was concave in pores and MPD monomer diffused more slowly out of the pores when contacting with TMC solution. Therefore, the hydrophilic pore wall limited the violence of the initial MPD “eruption” and gave rise to more PA formation deeper within support layer pores result in the overall path length for water and solute transport increased. In supports with hydrophobic surface, less MPD solution permeated in pores and the aqueous MPD solution meniscus was convex in pores, and the initial MPD rapid “erupted” to form pores, thus led to thicker, rougher PA film when contacting with TMC solution. Ghosh *et al.*^26^ discussed the properties of PA films formed *via* IP conditions over porous PSF supports with different physical and chemical properties. The investigation indicated that in support with hydrophilic pore walls, more PA formed inside pores, whereas for the relatively hydrophobic supports more PA formed above the pores. Therefore, the relatively hydrophilic supports produced slightly thinner, smoother, less permeable PA film, while the relatively hydrophobic supports produced slightly thicker, roughness, more permeable PA film, as illustrated in Fig. 6. The initial stages of the IP reaction are pictured as “volcano-like”, where MPD erupts from within the support membrane skin layer pores. The nearly instant reaction with TMC in hexane, initially, small nuclei of polyamide material suspended above pore openings. As MPD continuously erupts from the pores and partitions into the organic phase, it diffuses laterally to create a continuous polyamide film across the regions spanning pore openings. The lateral film growth connects the initial tufts, which are also continuously growing. The tufts reach a higher molecular weight than the laterally spreading base layer, which gives rise to the rugose morphology characteristic of thin film composite membrane. Some papers indicated the hydrophilicity of supports affected the connection of active layer and support layer, relatively hydrophilic surface facilitate enhance the connection of two layers and less possible to delamination.

### 3.4 The effect of additive on IP

Although the physicochemical properties, pore structure, roughness of support significantly dominate the performance of PA film, we shall not ignore the role of related additives in IP process. Relatively limited researches have been published on the role of additives in formation of PA TFC membranes in recent years. Sodium dodecyl sulfate (SDS) and trihexylamine (TEA) are the commonly used additive in IP MPD and TMC on polymer ultrafiltration support process. SDS works as a strong anionic surfactant, in some cases. It is used to improve wettability of the top surface of the support layers and amide solution effectively wetting support surface pores. Further to improving polymerization efficiency, because of wetted surface helping monomer in aqueous solution move into the organic solution and PA layer uniformly coated on support surface. The moderate amount of SDS can improve the related properties of composite membrane. However, the excess amount of SDS will cause the presence of defective the PA layer, due to the excess amount of SDS producing a number of free bodies on the PA film, which are easily fall off from the PA film after the polymerization reaction.^26,52,53^ Besides the commonly used surfactant of SDS, there are some others surfactants also used in IP, such as cationic surfactant cetyl trimethyl ammonium bromide (CTAB), non-ionic surfactant Trition X-100 and trimethyl benzyl ammonium bromide (TMBAB) and triethyl benzyl ammonium chloride (TEBAC).^54,55^ Mansourpanah *et al.* investigated the effects of addition of CTAB, SDS and Trition X-100 surfactants in the organic phase for preparing the NF membrane composite. The membrane using SDS showed the highest flux and rejection, and the membrane containing Trition X-100 showed a moderate rejection and diminished the flux, with a 90% rejection of Na_2_SO_4_, 70% of NaCl and 50% of MgCl_2_, and respectively, are the result of CTAB.

The TEA in the MPD aqueous solution is an acid equilibrium accelerating the MPD and TMC reaction, due to that the hydrogen chloride (HCl) is formed in IP. It is known that neutralizing HCl produced during amide formation is beneficial to the processing of the positive reaction of the chemical equilibrium.^56^ In addition, the addition of TEA can adjust the PH of solution, and the PH of aqueous amide solution has an important impact on the final performance of the TFC membrane.^57^ In general, the addition of the appropriate TEA was expected to promote the cross-linking and PA active layer formation. However, in some cases, the addition of TEA tends to reduce the salt rejection of the finial TFC membrane. This may be because TEA addition could compete with MPD portioning and diffusion that interrupted the polymerization reaction and the film formation, resulting in non-uniform PA layer formation with a large standard deviation.^31^

### 3.5 Porous support surface modification

Recently, there are some investigations to explore a variety materials of support membrane, but till now, the PSF microfiltration membrane is still a preferred membrane material, due to the fact that it is relatively hydrophilic and can easily be used for soaking in aqueous amide solution compared with others polymers, such as PP, PE, PVDF, PTFE and PVC *etc.*, even when PSF is not inherently hydrophilic polymer. Expanding the selection of supports is expected to enhance the performance and other properties of the TFC membrane. However, in order to make these polymers applicable to produce support layer of the TFC membrane, many studies have attempted to improve the performance of membrane surfaces using various techniques. Membrane surface modification has become one of the most important fields in the research. The techniques commonly used in the modification are physical blending, chemical grafting, and surface chemical reaction. Among them, improving the hydrophilic of surface by physical blending and chemical grafting methods are the most effective methods. Chemical grafting mainly introduces some hydrophilic groups on the surface of the polymeric membranes, for example, hydroxyl, carboxyl and amine group. These two methods not only improve the hydrophilic of membrane surface, but also optimize the pore structure of membrane, further affecting the performance of PA thin film.

#### 3.5.1 Organic monomer blending and grafting modification

**Fig. 7:**
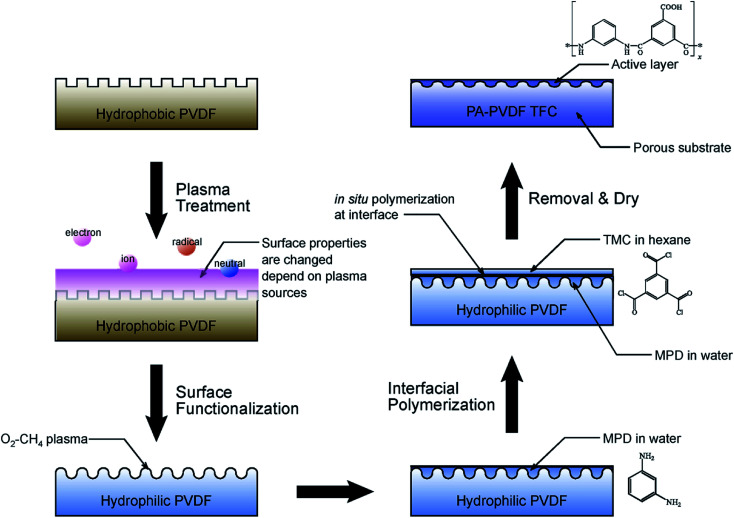
Schematic diagram of experimental process of plasma treatment and interfacial polymerization.^60^

Chemical modification has been widely utilized in improving the surface properties of polymers, including chemical grafting, polymerization, and plasma treatment.^24,30,58–65^ Among these methods, plasma treatment technique is the most effective method to graft organic monomers onto the membrane surface. It has a lot of advantages including fast production, simplicity and economy, as well as minimum effect on the bulk properties of materials. Plasma treatment can graft some hydrophilic monomers or producing some hydrophilic groups onto the support membrane surface and change the initial physicochemical properties of the membrane. Low temperature plasma treatment of PP and PSF support membranes was carried out by Kim *et al.*^66^ to investigate the performance enhancement of the TFC-RO membrane. The results showed the plasma treatment of membrane supports assisted in better interfacial polymerization to enhance the adhesion properties between the active layer and the support. In addition, the chlorine resistance of composite membrane also was enhanced due to produced chemical bonding (hydrogen bond and covalent bond) between the active layer and the support layer. The PVDF microporous membrane as the support layer of TFC membrane has been hindered by the hydrophobic nature. In order to improve the hydrophilic of PVDF, Kim *et al.*^60^ utilized oxygen, methane and their 1 : 1 mixture gas by plasma treatment technique to modify the PVDF membrane. The experiment results demonstrated PA-PVDF composite membrane better enhanced the water permeability and rejection than PA-PSF membrane. Fig. 7 depicted the schematic diagram. Suzuki^67^ group investigated the impact of plasma modification on PAN UF membrane characteristics. The results indicated the plasma treatment enhanced the membrane surface hydrophilicity and membrane permeability, and plasma polymerization reduced the membrane surface pore size and increased the rejection of membrane. Hydrophilic monomers such as acrylic acid, acrylonitrile, allylamine, ethylenediamine and *n*-propylamine were used to hydrophilize the support membrane.

Organic monomers blending and chemical coating are also effective methods to improve the properties of membrane surface, not only increasing the hydrophilic of surface, but also adjusting the pore structure and morphology of membranes. Incorporating hydrophilic monomers into hydrophobic supports has been reported in the published literatures, utilizing the related properties of monomers to optimize the water permeability, selectivity and stability of TFC membrane. Polyvinyl alcohol (PVA) is a well-known material that has been used in membrane modification due to its highly solubility in water, chemical resistance, highly hydrophilicity, as well as a environmentally friendly material, but PVA must be cross-linked by another material to reduce its water solubility. Park *et al.*^68^ utilized PVA to improve the hydrophilicity and mechanical strength of PVDF nanofibers membrane *via* dip coating and cross-linking with glutaraldehyde. The fabricated PVA-modified TFC membrane exhibited high hydrophilicity, mechanical strength and excellent water flux. Corvilain *et al.*^69^ added a hydrophilic polymer sulfonated polyether ether ketone (sPEEK) to the PSF membrane to optimize the support layer of the TFC membrane, and the bottom surface porosities and pore size were simply controlled by adjusting the polymer blend ratios. Zhu *et al.*^70^ blended conducting polymer polyaniline (PANI) into the PES casting solution to fabricate a PES/PANI blended membrane, PANI as both pore forming agent and hydrophilic modifier during UF membrane formation, resulting in the increase of membrane pore size, porosity and hydrophilicity, when PANI content is 0.2 wt% it could greatly enhance the flux and rejection of TFC membrane. Yao *et al.*^42^ blended amphiphilic copolymers of methyl methacrylate and 2-hydroxyethyl methacrylate (P(MMA-*co*-HEMA)) into the PVC substrates and the reactable groups were produced. The TFC membrane formed thereon displayed higher NaCl and Na_2_SO_4_ rejection and adequate adhesion compared to the control membrane.

#### 3.5.2 Inorganic nanoparticles blending modification

In past few decades years, the booming of nanotechnology that leads to some special performance and unique structures of nanoparticles, gradually attracted researchers attention. Incorporating nanoparticles into membrane materials has been considered as a way to make polymeric membranes more attractive to be commercialized, because of nanoparticles improving the related properties of the membrane, such as flux, the fouling resistance, mechanical strength, thermal stability and *etc.* Recently, a number of previous studies focused on modifying the TFC membrane support layer *via* incorporation of hydrophilic nanomaterials such as titanium dioxide (TiO_2_),^35,71–73^ graphene oxide (GO)^74,75^ or modified graphene oxide, modified carbon nanotubes (CNTs),^63,65,76–81^ silica nanoparticles,^82^ and porous zeolite nanoparticles.^83,84^ They are classified into zero-dimensional (0D) fillers, one-dimensional (1D) fillers and two-dimensional (2D) fillers.

##### 3.5.2.1 Zero-dimensional (0D) fillers

Zeolites were successfully utilized in chemical industry for several years. Recently it also has been introduced in membrane separations due to the superior adaptability, such as the high regularity, well-defined pore structure and excellent stability. The addition of the zeolites into membrane matrix has been found to grant nanocomposite membranes higher permeation flux and solute rejection compared to the pristine membrane. PVDF is an intrinsic hydrophobic polymer. In order to increase the water permeability of PVDF membrane, Yan *et al.*^85^ incorporated hydrophilic nano-sized alumia (Al_2_O_3_) uniformly in PVDF doping solution to increase the permeation-flux and anti-fouling performance, as well as the tensile strength and break elongate ratio of membrane, because of adding Al_2_O_3_ nanoparticles into membrane increased the surface hydrophilicity and the efficient filtration area, but did not affect the pore size, and porosity of PVDF membrane. Emadzadeh *et al.*^73^ investigated the effects of TiO_2_ nanoparticles (ranging from 0 to 1 wt%) loading on the properties of the PSF support and further studied how the changes in the support properties affected the performance of TFC membrane in the FO process by characterizing its hydrophilicity, overall porosity, surface roughness and morphology. Results showed that the hydrophilicity and porosity of the PSF membrane increased when TiO_2_ incorporated into the membrane, and a lower contact angle and a greater porosity value were obtained with a higher the TiO_2_ loading. However, the rejection of TFC membrane did not continuously increase with the higher the TiO_2_ loading. A greatest rejection was obtained when the concentration of TiO_2_ reached 0.5 wt%, due to the highest degree of cross-linking formed in the PA active layer. Ma *et al.*^86^ firstly reported that polysulfone-nanocomposite substrate membranes were prepared *via* phase inversion by incorporating porous zeolite nanoparticles in polysulfone. The PSF substrate with 0.5 wt% zeolite loading showed improved surface porosity and hydrophilicity. The TFC membrane preparing from such a substrate membrane showed significantly enhanced water permeability compared to the TFC membrane prepared on a conventional PSF substrate. Nevertheless, the authors observed that when zeolite loading was further increased to 1.0 wt% in the substrate, the integrity of polyamide rejection layer formed in the subsequent IP process was compromised. The author attributed this to some localized defects presenting in the PSF substrate membrane surface with 1.0 wt% zeolite loading, resulting in formation of an ineffective PA active layer.

##### 3.5.2.2 One-dimensional (1D) fillers

Carbon nanotubes (CNTs) have gained more attention because of the superior properties, such as high flexibility, low mass density, very simple chemical composition and structure, the remarkable atomic scale smoothness, and chemical inertness of their graphitic walls. In addition, CNTs possess the unique structure and performance, for example, the nanometer sized diameter, the hydrophobic inner pore wall, and an ultrafast water transport, as well as the easily functionalized CNT tip can be a facile target for localize chemical modifications to create a selective gate analogous to biological selectivity filters. CNTs have promising properties for water treatment, in mitigating membrane fouling through the inhibition of bacterial growth, and in improving the separation selectivity due to their narrow pore size distribution.^87^ In some literatures, the increments in special parameters like modulus and tensile strength, water flux and permeability, and rejection have been studied by adding CNTs to polymeric membranes. However, how to effectively dispersion and dissolution of synthesized CNTs in various organic solutions and different polymers and improving the weak interaction of the interface between the CNTs and polymer membrane are important problems that we faced. The efficient utilization of CNTs in composite applications depends on the ability to disperse the CNTs homogeneously throughout the support membrane and the compatibility between CNTs and the polymer membrane. Thus, in order to improving the dispersion capability, surface functionalization modification is the most effective method. Surface oxidation and introduction of hydrophilic functional groups into the surface of the CNTs can also be helpful to obtain a better dispersion of CNTs into relevant matrices. Son *et al.*^88^ successfully synthesized the TFC membrane with a functional CNTs/PES support membrane by phase inversion method and IP, and the TFC membrane showed enhanced water due to its increased hydrophilicity, enhanced pore properties of the support layer without sacrificing NaCl rejection compared to that of the TFC bare membrane. In addition, the TFC membrane exhibits a higher organic fouling resistance due to more negative charged surface. Celik *et al.*^77^ synthesized multi-walled carbon tube (MWCNTs) and PSF blend membranes and then investigated the anti-fouling efficiency and protein fouling behavior respectively. The results exhibited that foulants on the MWCNTs/PSF blend membranes surface could be more easily removed by caustic cleaning than on the bare PES membrane, resulting in higher flux recoveries with C/P blend membranes. In addition, The total protein fouling and the irreversible fouling ratio of the C/P composite membranes were less than the bare PES membrane for both bovine serum albumin (BSA) and ovalbumin (OVA) and the flux recovery ratio of the composite membranes was higher.

Halloysite nanotubes (HNTs) are a promising inorganic material that possess unique structure and special performance, such as nano-tubular, regular open-ending pores and a great deal of hydroxyls on their surface. Zhu *et al.* grafted sodium 4-styrenesulfonate onto the surface of HNTs *via* surface initiated atom transfer radical polymerization, and then blending them into PES membranes to fabricate NF membranes. The effects of composites on the PES membrane surface hydrophilicity, microstructure and charged capacity were investigated in detail. The results exhibited that the hydrophilicity and the water flux of blend membrane were enhanced significantly, as well as enhanced fouling resistance to a certain extent.

##### 3.5.2.3 Two-dimensional (2D) fillers

The recent advances in the microporous membrane materials revealed several interesting class of 2D fillers, such as ultra-thin graphene, graphene oxide and derivate, due to their outstanding mechanical and chemical stability.^89,90^ Incorporation of GO and derivate into support layer of TFC membrane could improve membrane hydrophilic and porosity, which enhanced the water permeability. Besides, functionalized support layer could affects the mechanical strength and the performance of the active layer composite membranes. Li *et al.* systematically investigated the effect mechanisms of GO on the membrane support layer for improving the water flux of TFC membrane. The results showed that the membrane of support was modified by GO possesses a higher water flux, because of GO could improve the porous structure and porosity. Wu *et al.*^91^ firstly synthesized SiO_2_-GO nanohybrid, in which silica densely and uniformly dispersed on the GO surface, giving rise to high hydrophilicity of SiO_2_ is helpful to homodisperse. They then prepared SiO_2_-GO/PSF blend membrane by SiO_2_-GO doping. The results exhibited that the incorporation of appropriate amount of SiO_2_ nanoparticle into the PSF matrix improved the substrate wettability and reduced *S* parameter of TFC membrane, leading to a 40% improvement of water flux. Furthermore, the hybrid microporous membrane that was developed by doping SiO_2_-GO nano hybrid exhibited nearly 2-fold increment in pure water flux with the rejection rate of albumin maintained at 98%.

## 4. Conclusion and prospects

The structure and performance of support membranes have crucial influence on the PA layer formation, and further affect the properties of TFC membrane. Selecting an appropriate support membrane material, as well as optimizing and adjusting the structure, can effectively improving the performance of TFC membrane and expand application fields. Optimizing the impact factors of support layers is a very complicated process. It includes a variety of factors (pore size, porosity, hydrophilicity and hydrophobicity, surface roughness, layer structure, polymer types and interfacial interaction). Each factor not only can be independently manipulated to enhance the overall membrane performance, but also can be synergistic effects to maximize the overall membrane performance. In this review, each impact factor was concluded and discussed in detail, in order to provide the readers a fundamental and comprehensive knowledge of support role on fabricate TFC membrane. In addition, we also have listed some modification methods respecting to support membranes by a large number of related literature summary in order to provide an insight for future development of support membranes.

The research trends of the support layer of TFC membrane mainly focus on two aspects: (1) the research of appropriate membrane material and formation mechanism; (2) optimizing and adjusting the structure and morphology of support membrane to enhance the functionality and durability of membrane materials. Continuous improvements in TFC membrane performances with respect to permeability, selectivity and stability perhaps in the future will widen the applications of membranes to new areas.

## Conflicts of interest

There are no conflicts to declare.

## Supplementary Material
